# Insights of window-bsed mechanism approach to visualize composite bioData point in feature spaces

**DOI:** 10.5808/GI.2019.17.1.e4

**Published:** 2019-03-31

**Authors:** Mosaab Daoud

**Affiliations:** 1 Department of Mathematics and Statistics, York University, Toronto, ON M3J 1P3, Canada; 2 Sanofi Pasteur (Industrial Partner), Toronto, ON M2R 3T4, Canada

**Keywords:** composite biodata point, diversity, Mosaab-metric space, segmented genetic variations, se­riousness of the difference, variants, visualization, window-based mechanism

## Abstract

In this paper, we propose a window-based mechanism visualization approach as an alternative way to measure the seriousness of the difference among data-insights extracted from a composite biodata point. The approach is based on two components: undirected graph and Mosaab-metric space. The significant application of this approach is to visualize the segmented genome of a virus. We use Influenza and Ebola viruses as examples to demonstrate the robustness of this approach and to conduct comparisons. This approach can provide researchers with deep insights about information structures extracted from a segmented genome as a composite biodata point, and consequently, to capture the segmented genetic variations and diversity (variants) in composite data points.

## Introduction

In recent years, biodata mining has new research problems that are related to the concept of composite data points. A data point is said to be a composite data point when it is a dataset; in other words, when it has a number of biosequences or data-vectors. The composite data point is a new generalization to the concept of the data point from the ordinary definition (e.g., a biosequence or a data-vector). In case of visualizing a composite data point using window-based mechanism, few technical problems arise in this context. In this paper, we shall address those technical problems and provide insights about the window-based mechanism visualization approach. In the next part of this section we shall present a summary of recent related research work. We will focus on the research achievements in the area of alignment-free sequence analysis.

Alignment-free sequence analysis is a developing research area [1, 2], and recent years have shown this scientific fact clearly. Alignment-free sequence analysis algorithms (AFSAA) have several strengths compared with alignment-based sequence analysis algorithms (ABSAA). First, AFSAA can be used to map sequences into feature space as data-vectors; therefore, several algorithms, techniques, and approaches in data mining, machine learning, and statistical computing can be implemented effectively to analyze data-vectors that are extracted from sequences in feature space. Second, AFSAA are computationally less expansive compared with ABSAA [3]. AFSAA are window-based algorithms. Those algorithms are applicable to any sequences [3] without any prior assumption about degrees of dissimilarities; in other words, prior knowledge about homology assumption is not required. Moreover, AFSAA can be used when ABSAA are inapplicable.

Daoud [4] proposed an alignment-free sequence analysis technique to measure the distance between two unaligned biosequences. The technique has the capacity to measure the distance between two sliding segmented windows. Quantum of distance values are concluded after sliding a segmented window on the longest sequence from left-end to right-end. The whole shortest sequence is considered as another static segmented window. The distance distribution is used to analyze the quantum of distance-values. The membership value of a given query sequence with respect to different classes can be estimated using stochastic approximation, and without assuming any prior stochastic assumptions.

Pham and Zuegg [5] proposed an alignment-free probabilistic measure to measure the distance between two unaligned sequences. Precisely, the probabilistic measure is defined to measure the distance between two estimated Markovian models, where each Markovian model represents a sequence. The research addressed the problems of alignment based-algorithms in terms of aligning sequences with low similarity rates and the time complexity to accomplish the required computational process.

Borozan et al. [6] proposed another approach to improve the classification outcomes of sequence comparison by combining alignment-free and alignment-based measures to obtain similarity scores as discriminatory information about sequences.

Vinga and Almeida [7] reviewed the majority of overwhelming alignment-free sequence comparison algorithms. The paper classified those algorithms into two categories. The first category is defined in terms of the frequency distribution of *n*-grams and the distance/similarity measures are defined in a feature space (Cartesian space). In this context, the data-vectors are the frequency vectors of *n*-grams. The second category is based on the implementation of Kolmogorov complexity and Chaos Theory.

The structure of this paper can be summarized as follows: we present the proposed method in section II; experiments and discussions are presented in section III; and finally, the conclusion and the expected future work are presented in section IV.

## Methods

**Fig. 1.**
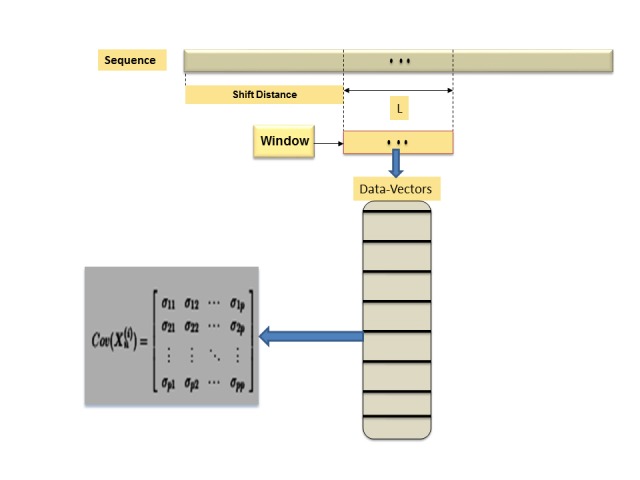
Computational process of window-based mechanize.

**Fig. 2.**
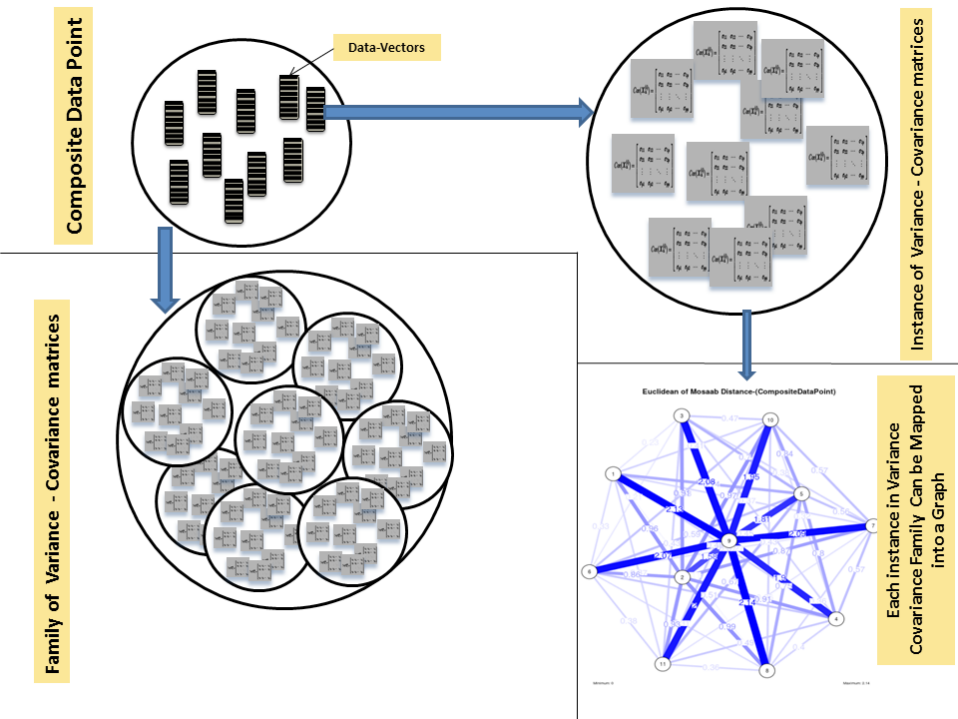
Prototypes for a composite data point, a family of variance-covariance matrices, an undirected graph model for an instance of variance-covariance matrices.

The window-based mechanism is a well-known mechanism in data science and biodata mining. Usually, it can be implemented with data that has a sequential relation to capture the local statistical parameters and to infer the main global information structure. The window-based mechanism has specific computational parameters, these parameters can be summarized as follows: (1) window-length or size (*L*), (2) shifting distance (*α*), and (3) random feature vector (*X_n_*). Those computational parameters play a key role in estimating the local statistical parameters and in inferring the main global information structure of the data under consideration. Therefore, those computational parameters provide the analyst with various insights about data, and they can help to understand data and to evaluate the implemented computational mechanism. In case of composite data point, each data point is a dataset, thus, we have another level of computations. In other words, we have to model data-insight of extracted information using a distance measure/metric or a composite distance measure/metric and a visualization tool (e.g., graphs). In case of a segmented genome of a virus, each segment can be encoded to 1 or more proteins, and each protein is a sequence. Therefore, a segmented genome is a composite data point. Now, without loss of generality, let us assume that we define a (*p*×1) random feature vector *X* in IR^p^, to use it in screening a composite data point and model its information structure. However, to model any information structure, we have to define a statistical concept, and in this case, we choose the variation theory as a statistical concept. In other words, we aim to model variation-based information structure as data-insight to evaluate the window-based mechanism and to visualize composite data point in a given feature space. In case of univariate or multivariate, the variation theory has various statistical parameters and models. One of those parameters is the variance-covariance matrix. Define *X_n_* as occurrence of all possible *n*-grams, hence *X* has the dimension (*p*×1), where $p=|\Sigma |{\;}^{n}$, Σ is a finite alphabet, and *n* is string-length. Define the variance-covariance matrix of *X_n_* as Ω*X_n_*, which it has the dimension (*p*×p). Up to this point, each sequence in a composite data point can be represented by a variance-covariance matrix. Thus, let the composite data point be denoted by *CDP = {Seq_1_, Seq_2_, ..., Seq_m_}*, thus, obviously each sequence *Seq_i_* in *CDP* can be represented by a variance-covariance matrix Ω*X_n_* . To compute Ω*X_n_* , we have to extract data-vectors *{x_1_, x_2_, ..., x_l_}* from *Seq_i_* as defined by *X_n_* using the window-based mechanism. We can motivate the main idea of this paper in the following way. We aim to model an existing information structure as a data-insight of a given composite data point using undirected graph as a visualization technique, and to evaluate window-based mechanism as a feature extraction technique. One of the essential difficulties involved in this problem is measuring the distance between any two variance-covariance matrices. As stated in his PhD dissertation, Mosaab Daoud [8] proposed a solution for the composite data points proximity problem. The solution defined a new metric space $\left(\Psi ,Dij\left(\gamma 1\right)\right)$, where Ψ is a class of composite data points, and *D_ij_*(*γ*1)is a metric. *D_ij_*(*γ*_1_) is defined as follows:

$$D_{ij}(\gamma 1)\;=\;|\gamma_{1}^{j}\;({Ω}_{X_{n}}^{\left(i\right)}\;-\;{Ω}_{X_{n}}^{i}\;)\gamma 1|\;=\;|\lambda 1|\;>\;0$$

where λ_1_ is the largest generalized eigenvalue (associated with the generalized eigenvector *γ*1) of the matrix $\left({Ω}_{X_{n}}^{\left(i\right)}-{Ω}_{X}^{\left(j\right)}\right)$. Now, by using the window-based feature extraction mechanism we can map *CDP* into a family of sets of data-vectors $DV_{\alpha ,L}=\{x_{1}^{\left(i\right)},\;x_{2}^{\left(i\right)},\;...\;,\;x_{l}^{\left(i\right)}\};\;S_{\left(i\right)}^{e}q_{i}\in CDP,\;\alpha \in \Theta ,L\in Z^{+}\}$ thereafter, we can map each *DV_α,L_* into a set of variance-covariance matrices $VC=\{{Ω}_{X_{n}};Seq_{i}\in CDP\}$. In this way, we compose a family of sets of variance-covariance matrices. By implementing the metric *D_ij_*(*γ*_1_), we can map each *V C* into a set of distance values in the interval [0, ∞). It should be noted that Θ is a set of all possible values of the shifting-distance *α, L* is the window-size, and Z^+^ is the set of positive integers (Θ and Z^+^ are parameter spaces). In decision theory and risk analysis [9], we have a concept called the seriousness of the difference. It is hard to measure the seriousness of the difference in a family of variance-covariance matrices (note: a family is a set of instances, and each instance is a set of variance-covariance matrices), but we can depict the seriousness of the difference among instances in a family of variance-covariance matrices using undirected graph, which is one of the objectives of this paper. Consequently, in an undirected graph, each sequence will be represented by a node and each distance value will be represented by an edge. In this way, we can measure the seriousness of the difference in a family of variance-covariance matrices. Finally, in the next section, we shall discuss the proposed approach by using real data. The computational process of this approach is illustrated in Figs. 1 and 2.

## Results and Discussion

In this section, we shall present the implementation of the proposed approach using real data. Meanwhile, we shall discuss the practical outputs and implementations in details. We will use the segmented genomes of flu virus, and segments of Ebola virus as composite data points.

One of the highly mutable viruses is the flu virus, and it has serious negative impacts on various populations (e.g., human population). The genome of influenza virus has eight segments, and each segment can be encoded into either 1 or 2 proteins. The virus is classified under the family Orthomyxoviridae [10-12]. The eleven RNA-proteins of influenza virus genome are: PB1 (polymerase protein), PB2 (polymerase protein), PA (polymerase protein), HA (haemagglutinin protein), NP (nucleoprotein), NA (neuraminidase), M1 (matrix protein), M2 (matrix protein), NS1 (non-structural protein), and NS2 (non-structural protein). The variability of the influenza virus is embedded in the genetic text of the two surface proteins: (1) HA and NA [13, 14]. The identification of influenza sub-type can be accomplished using the variability of HA and NA proteins.

The other composite data point that we shall consider in this paper is Ebola virus. The Ebola virus is a negative-sense RNA virus, and it is classified under the family Filoviridae [15]. The genome of Ebola has seven segments. The seven RNA proteins of Ebola virus genome are follows: nucleoprotein (NP), nucleocapsid protein (VP35), matrix protein (VP40), glycoprotein (GP), nucleocapsid protein (VP30), nucleocapsid protein (VP24), and polymerase protein (*L*).

**Fig. 3.**
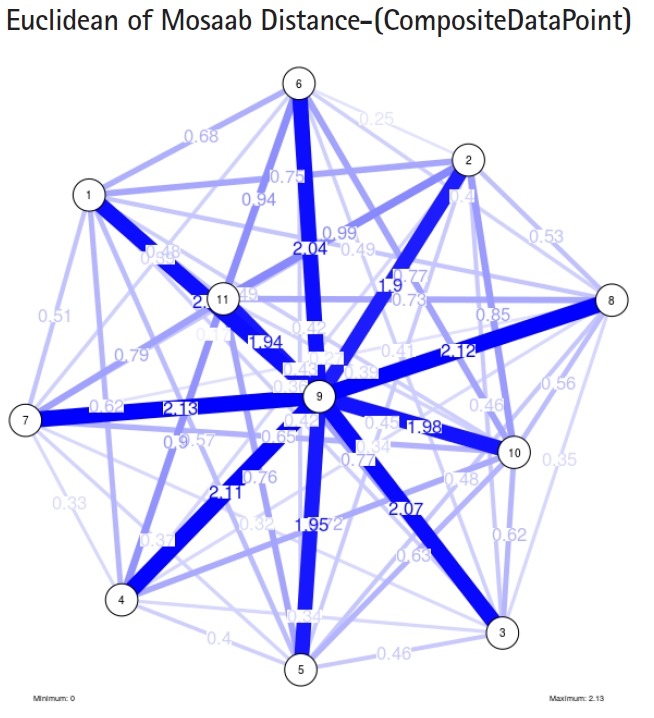
The insight of information structure for Influenza A virus. This instance of variance-covariance matrices is captured using the following parameters: *α* = 1, *L* = 50, and *n* = 1.

**Fig. 4.**
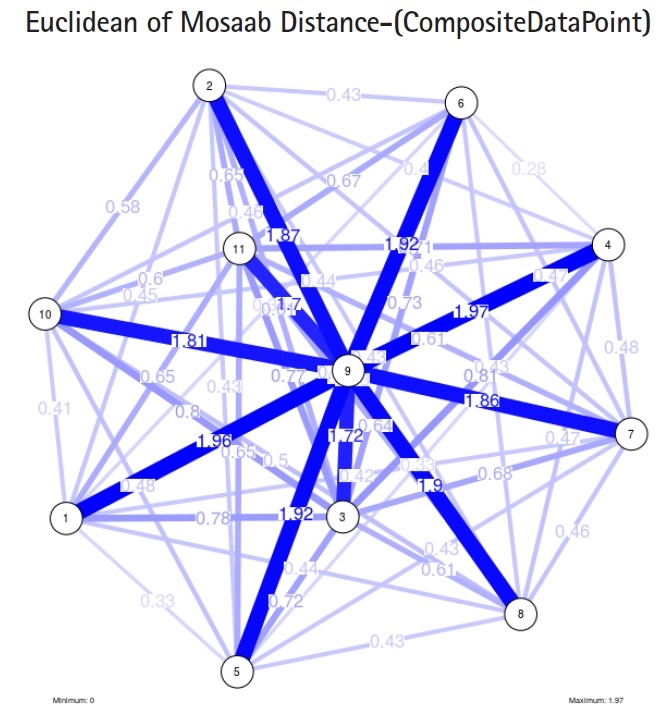
The insight of information structure for Influenza A virus. This instance of variance-covariance matrices is captured using the following parameters: *α* = 1, *L* = 50, and *n* = 2.

**Fig. 5.**
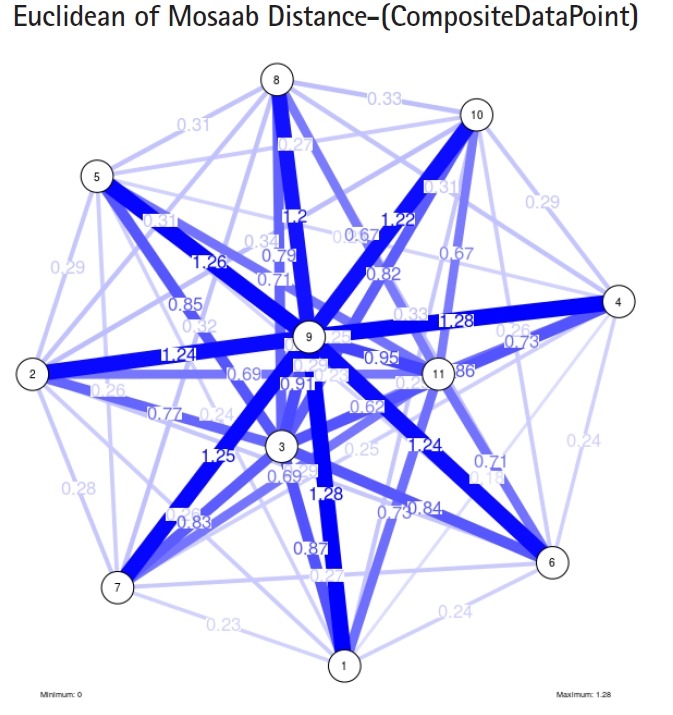
The insight of information structure for influenza A virus. This instance of variance-covariance matrices is captured using the following parameters: *α* = 1, *L* = 50, and *n* = 3.

**Fig. 6.**
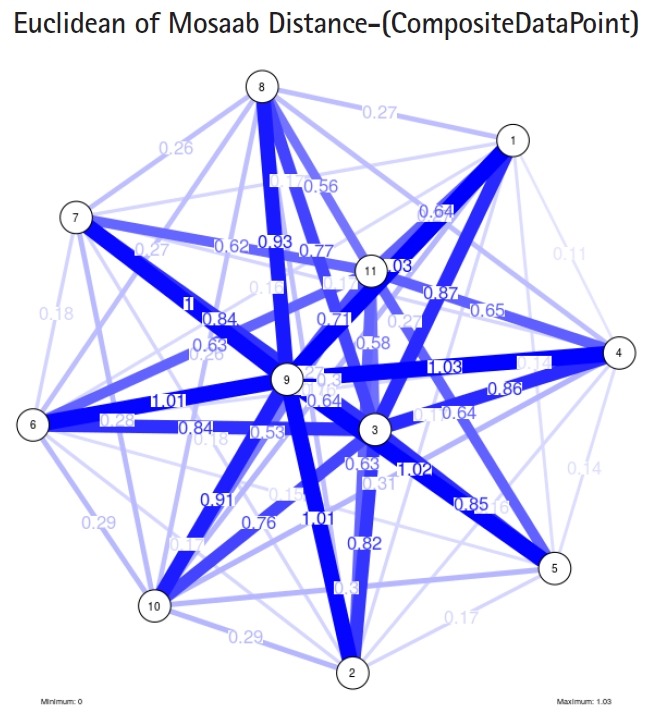
The insight of information structure for influenza A virus. This instance of variance-covariance matrices is captured using the following parameters: *α* = 1, *L* = 50, and *n* = 4.

To proceed further, we downloaded a few composite data points from on-line databases. The composite data points represent the segmented genomes of influenza virus type A, influenza virus type B, and Ebola virus. Consequently, we compose a family of variance-covariance matrices for the composite data point: influenza virus type A, which it has 4 instances: the first instance: *α* = 1, *L* = 50, and *n* = 1; the second instance: *α* = 1, *L* = 50, and *n* = 2; the third instance: *α* = 1, *L* = 50, and *n* = 3; and the forth instance: *α* = 1, *L* = 50, and *n* = 4. Each instance represents an information structure of the composite data point, and each undirected graph represents an insight of the information structure. To measure the seriousness of difference in a family of variance-covariance matrices, we depict those instances in Figs. 3–6. From those figures we can conclude the seriousness of the difference caused by considering different feature vectors. Fig. 3 has the highest variability (spread) compared with Figs. 4–6. In other words, in the graph, the distances among nodes can be used as an indicator about the seriousness of the difference caused by biodiversity and/or variability to detect new variants.

**Fig. 7.**
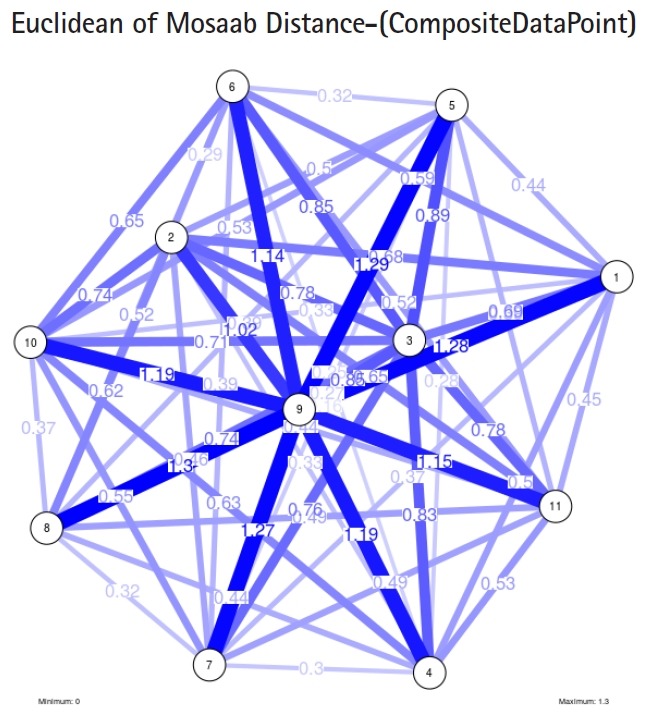
The insight of information structure for influenza A virus. This instance of variance-covariance matrices is captured using the following parameters: *α* = 1, *L* = 80, and *n* = 1.

**Fig. 8.**
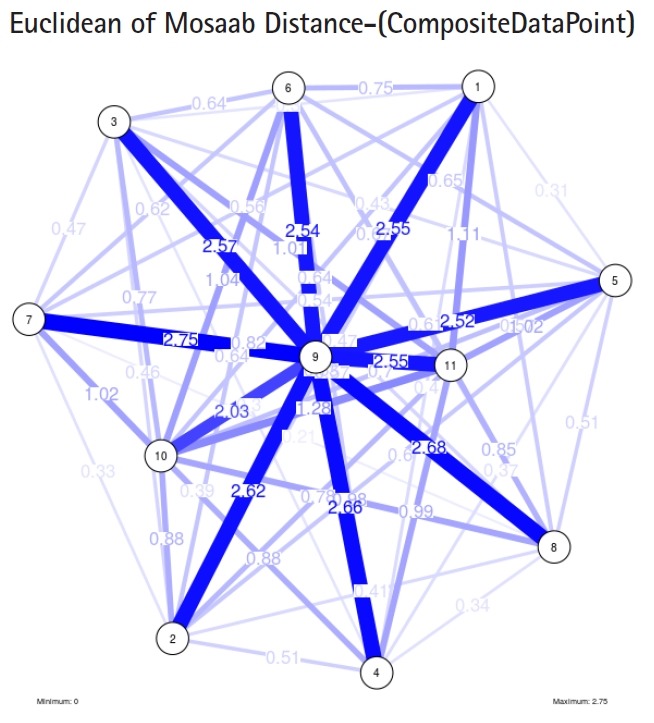
The insight of information structure for influenza B virus. This instance of variance-covariance matrices is captured using the following parameters: *α* 1, *L* = 80, and *n* = 1.

**Fig. 9.**
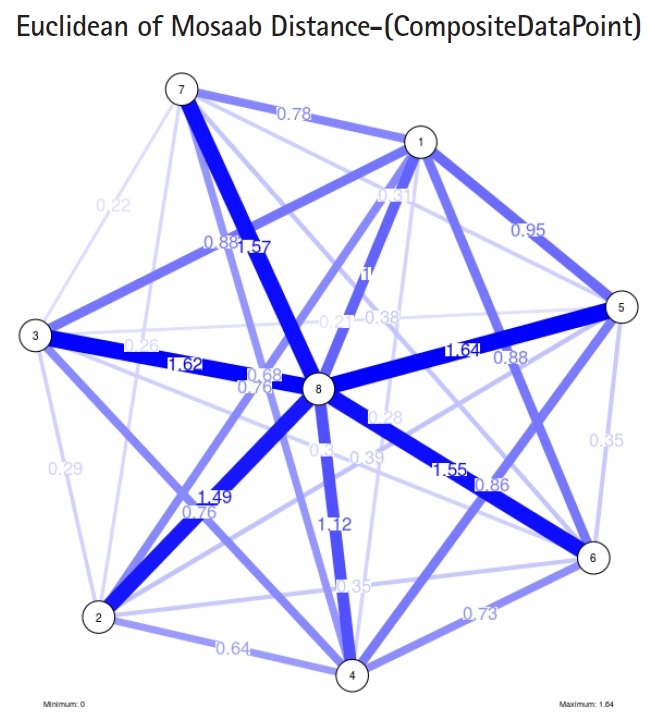
The insight of information structure for Ebola virus. This instance of variance-covariance matrices is captured using the following parameters: *α* 1, *L* = 80, and *n* = 1.

In the second part of this experiment, we compare three composite data points. Each composite data point is a segmented genome. Those composite data points are follows: (1) influenza virus type A, (2) influenza virus type B, and (3) Ebola virus. We compose a family of variance-covariance matrices with three instances using the following parameters: *α* = 1, *L* = 80, and *n* = 1. Figs. 7–9 depict those instances respectively. It is clear that the insights of inner information structure of those composite data points are different in terms of distance-variability and inner information structure, and this variability reflects the genetic diversity in the segmented genomes of the considered viruses.

Another comparison can be conducted between the two instances of variance-covariance matrices given in Figs. 3 and 7, and it is clear that the seriousness of the difference occurs between the two instances due to the window-size.

Based on these results, we should shift the purpose of this approach to the level of a tool. This tool can help researchers and users in the field of computational biology to understand and evaluate the mechanisms of window-based approaches, and to understand the segmented genetic variation of a composite data point through depicting the seriousness of the difference among information structures extracted from a given composite data point using window-based mechanism. In addition, the tool can be used to visualize the genetic diversity of composite data points.

We presented experiments and results, and in the next section, we shall present conclusions and future work.

In this paper, we have analyzed window-based mechanism approach as a sequence analysis approach. We introduced the terminology: the seriousness of the difference, composite data point, data insight, and information structure. There is a difficulty in measuring the seriousness of the difference among the existing insights of information structure in a composite data point. The contributions of this paper can be summarized as follows: we proposed the concept of a family of variance-covariance matrices, where each instance of this family is a set of variance-covariance matrices, which represents a data-insight about the information structure of a composite data point. We proposed an alternative approach to measure the seriousness of the difference among data-insights extracted from a composite data point by using undirected graph and Mosaab-metric space to visualize the differences caused by estimates of the parameters: (1) window-length or size (*L*), (2) shifting distance (*α*), and (3) random feature vector (*X_n_*). This approach can be used to evaluate window-based sequence analysis algorithms, and to capture segmented genetic variation and diversity in composite data points. The approach can be used to answer critical biological questions: for example, are the corresponding segments of influenza A and B similar in distance? Can we capture the rates of change in those segments? Which may be interesting to epidemiologists. Finally, as future work, we can use this approach as an integrated tool to visualize the diversity and variability of outliers (variants) in a dataset of composite data points.
